# Mechanisms of tRNA-derived fragments and tRNA halves in cancer treatment resistance

**DOI:** 10.1186/s40364-020-00233-0

**Published:** 2020-10-15

**Authors:** Yue Zhang, Huizhu Qian, Jing He, Wen Gao

**Affiliations:** Department of Oncology, The First Affiliated Hospital of Nanjing Medical University, 300 Guangzhou Road, Nanjing, 210029 China

**Keywords:** tRNA-derived fragments, tRNA halves, Cancer, Drug resistance, Biomarker

## Abstract

The tRNA-derived fragments (tRFs) and tRNA halves (tiRNAs) are newly discovered noncoding RNAs in recent years. They are derived from specific cleavage of mature and pre-tRNAs and expressed in various cancers. They enhance cell proliferation and metastasis or inhibit cancer progression. Many studies have investigated their roles in the diagnosis, progression, metastasis, and prognosis of various cancers, but the mechanisms through which they are involved in resistance to cancer treatment are unclear. This review outlines the classification of tRFs and tiRNAs and their mechanisms in cancer drug resistance, thus providing new ideas for cancer treatment.

## Background

Transfer RNAs (tRNAs) have long been regarded as classic noncoding RNAs and are involved in protein translation [1]. Recently, many researchers have discovered new noncoding RNAs that are derived from specific cleavage of pre- and mature tRNA. Noncoding RNAs derived from tRNA are grouped into two categories: tRNA-derived fragments (tRFs) and tRNA halves (tiRNAs) [1]. The tRFs originate from mature or pre-tRNAs, and they are approximately 14–30 nucleotides (nt) in length. tiRNAs are 29–50 nt in length and originate from specific cleavage of mature tRNA anticodon loop under stress [2, 3]. The tRFs are conservative and widespread in nature [4]. They were initially discovered as random tRNA degradation fragments and later found to be generated by conservative and specific tRNA cleavage [5]. The tRFs are associated with cancer, inherited metabolic diseases, viral infections, and neurodegenerative diseases [6]. Here, we introduce the classification of tRFs and tiRNAs, discuss their roles in cancers, summarize their mechanisms of drug resistance in cancer treatment, and describe techniques for studying tRFs and tiRNAs.

### Classification of tRFs and tiRNAs and their roles in cancers

**Fig. 1:**
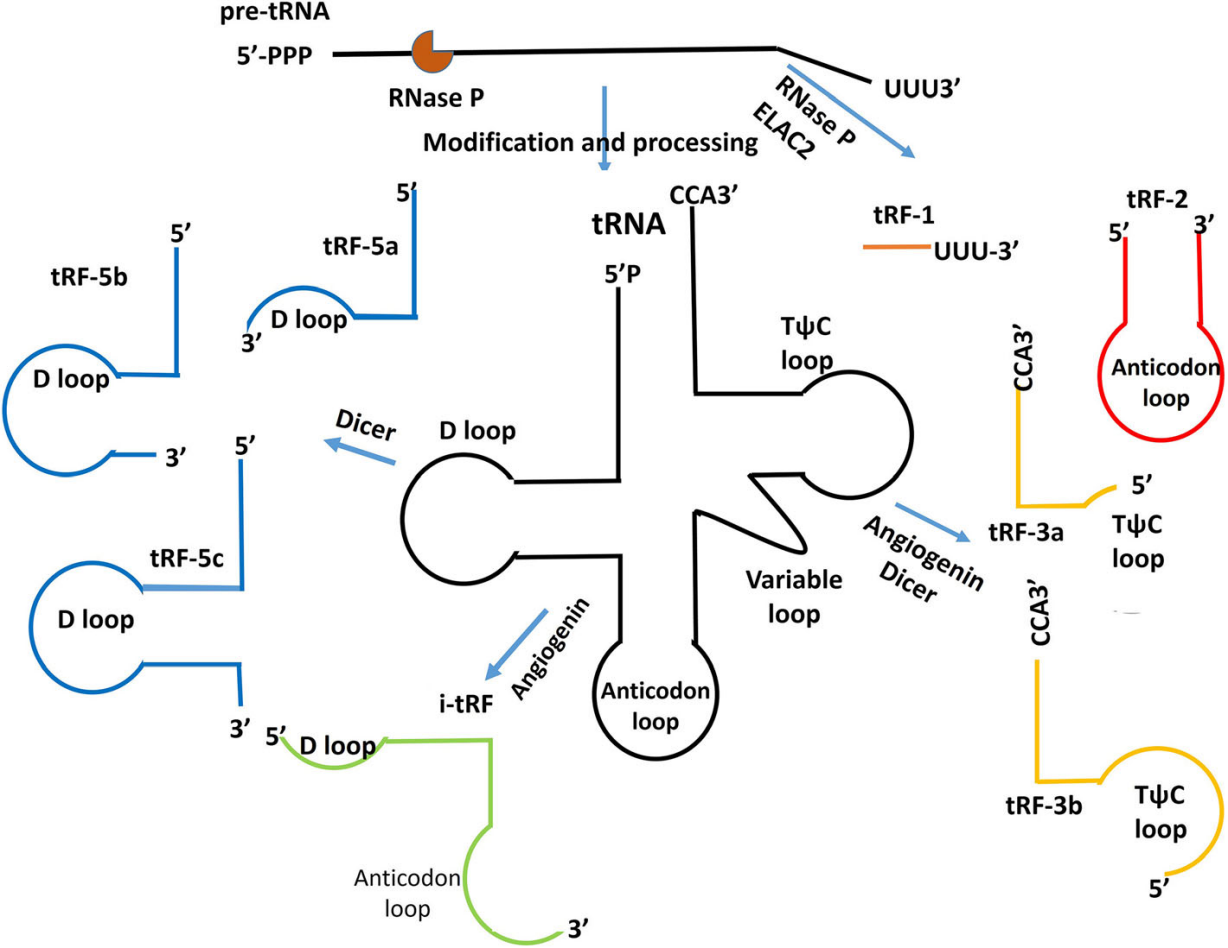
Classification of tRFs. TRFs include tRF-1, tRF-2, tRF-3, tRF-5, and i-tRF. tRF-1 is generated from the 3′-end of pre-tRNA. tRF-2 is a tRNA fragment containing an anti-codon loop generated by an unknown cleavage method. tRF-3 and tRF-5 are derived from the 3′- and 5′-ends of mature tRNAs, respectively. i-tRF originates from the internal region of mature tRNA

The tRFs include tRF-1, tRF-2, tRF-3, tRF-5, and i-tRF [5] (Fig. 1). The tRF-1 is produced by cleaving 3′ pre-tRNA by RNase Z or its cytoplasmic ribonuclease Z 2 (ELAC2) in the TψC loop [7]. tRF-1 has carcinogenic or anticancer effects in the occurrence and development of cancers [5]. tRF-2 is derived from the decomposition of anticodon loops of tRNAs under hypoxic condition [8]. Angiogenin and Dicer cleave the T-loop of 3′-ends of mature tRNA to produce tRF-3. tRF-3 includes tRF-3a and tRF-3b [7, 8]. Dicer cleaves the D-loop of tRNA to produce tRF-5. tRF-5 includes tRF-5a, tRF-5b, and tRF-5c [5]. The tRF-5 is mostly located in the nucleus, whereas tRF-3 and tRF-1 mainly occur in the cytoplasm [8]. The i-tRF spans the anticodon loop and is derived from mature tRNA [9]. The tiRNAs are produced by cleaving the anticodon loop of mature tRNA by angiogenin under stress conditions. There are two types of tiRNAs: 5′-tRNA half (tiRNA-5) and 3′-tRNA half (tiRNA-3) [10]. Nutritional deficiency, hypoxia, heat shock, and oxidative stress can stimulate angiogenin activity and increase tRNA cleavage [11].

The tRFs and tiRNAs regulate ribosome and protein biogenesis; DNA damage response; gene expression; RNA interference; immune mediation; and cell proliferation, apoptosis, and migration [4, 8, 57]. They are expressed and dysregulated in various cancers [58] (Table 1). The heterogeneity and stability of tRFs make it suitable biomarkers for cancer diagnosis and prognosis [19]. Acquired treatment resistance is the main dilemma of cancer treatment [59]. Many studies have systematically discussed the roles of tRFs and tiRNAs in the diagnosis, progression, metastasis, and prognosis of various cancers [1, 5], but few studies have investigated their roles in resistance to cancer treatment. Herein, we summarize their mechanisms in cancer drug resistance (Table 2).

**Table 1:** Roles of tRNA-derived fragments and tRNA halves in cancers

Cancer	tRFs and tiRNAs	Type	Findings	References
breast cancer	tRF-30-JZOYJE22RR33 tRF-27-ZDXPHO53KSN	tRF	Independent predictors of PFS in HER-2-positive breast cancer	[12]
	tRF-0009 tDR-7336	tRF	Facilitate doxorubicin resistance	[13, 14]
	tRF3E	tiRNA-3	tumor suppressor	[15]
	tDR-7816	tRF	diagnostic biomarker of early non-TNBC	[16]
	ts-112	tRF	ts-112 inhibition reduces the proliferative capacity of aggressive breast cancer cells.	[17]
	5′-tiRNA^Val^	tiRNA-5	5′-tiRNAVal overexpression significantly suppresses breast tumor cell proliferation, migration and invasion.	[18]
	tRF-32-Q99P9P9NH57SJ	tiRNA-5	tumour stage and lymph node metastasis	[19]
	SHOT-RNAAsp-GUC SHOT-RNAHis-GUG SHOT-RNALys-CUU	tiRNA	SHOT-RNAs enhance cell proliferation.	[20]
	tDR-000620	tRF	aggressive phenotype of breast cancer stem cells	[21]
	5′-tiRNA^Asp^, 5′-tiRNA^His^	tiRNA-5	elevate in breast cancer	[22]
	tRF^Glu-YTC^ tRF^Asp-GTC^ tRF^Gly-TCC^	i-tRF	suppress cell proliferation and cancer metastasis	[23]
	tRF^Ser-GCT^	i-tRF	Unknown	[24]
	tRF-2 derived from tRNA^Glu^, tRNA^Asp^, tRNA^Gly^, and tRNA^Tyr^	tRF-2	Bind to YBX1 by displacing 3′-UTR, thus suppressing cancer cell growth and metastasis	[25]
	ts-46, ts-47	tRF-1	ts-46 and ts-47 are upregulated by PIK3CA and KRAS mutations, respectively. These two mutations are involved in the resistance of breast cancer cells to lapatinib.	[26]
lung cancer	ts-101 ts-53	tRF-1	Associate with PiwiL2, an essential protein involved in silencing of transposons	[25]
	ts-46 ts-47	tRF-1	have inhibitory effect on the ability of lung cancer cells to form colonies	[25]
	tRF^Leu − CAG^	tiRNA-5	Promote cell proliferation and G0/G1 cell cycle progression	[27]
pancreatic cancer	AS-tDR-000064 AS-tDR-000069 AS-tDR-000102 AS-tDR-001391	tiRNA-5	diagnostic and therapeutic biomarkers	[28]
colorectal cancer	tRF/miR-1280	tRNALeu and pre-miRNA	suppress colorectal cancer growth and metastasis	[29]
	tiRNA-Tyr-GTA	tiRNA-5	Targets of tiRNA-Tyr-GTA are mainly enriched in the PPAR signaling pathway.	[30]
	tRF-Gln-CTG	tRF-5c	Negative regulation of c-Jun N-terminal kinase (JNK) cascade is enriched in tRF-Gln-CTG. Inhibition of JNK cascade can reduce the migration potential of colon cancer cells in vitro.	[30]
	tRF-Leu-TAG	tRF-5a	Function of mesenchymal-to-epithelial transition is enriched in tRF-Leu-TAG.	[30]
	5′-tRF-Lys^TTT^, 5′-tRF-Val^CAC^, 5′-tRF-Glu^CTC^, 5′-tRF-Pro^CGG^	tRF-5	Associate with chemotherapy treatment outcomes	[26]
prostate cancer	tRF-1001	tRF-1	TRF-1001 is required for cell proliferation.	[31]
	tRNA^Lys-CTT^ tRNA^Phe-GAA^	tRF	The ratio of tRFs derived from tRNA^Lys-CTT^ and tRNA^Phe-GAA^ is a good indicator of progression-free survival.	[32]
	tRF-544 tRF-315	tRF-5 tRF-3	High expression ratio of tRF-315/tRF-544 predicts poor PFS.	[32]
	SHOT-RNAAsp-GUC SHOT-RNAHis-GUG SHOT-RNALys-CUU	tiRNA	SHOT-RNAs enhance cell proliferation.	[20]
liver cancer	tRF^Ser^	tRF-3	Cleavage of tRNAs during stress	[33]
cervical cancer	tRF^Gln^	tRF-5	inhibit the process of protein translation	[34]
clear cell renal cell carcinoma	5′-tRNA4-Val-AAC	tiRNA-5	advanced stage and grade	[35]
	5′-tiRNA-Arg-CCT, 5′-tiRNA-Glu-CTC, 5′-tiRNA-Leu-CAG, 5′-tiRNA-Lys-TTT	tiRNA-5	Potential tumor suppressors	[36]
B cell lymphoma	CU1276 (tRF-3018)	tRF-3	CU1276 associates with argonaute proteins, represses endogenous RPA1, suppresses proliferation, and modulates molecular response to DNA damage. Loss of CU1276 expression may confer a growth advantage to malignant cells.	[37]
	tRFHis-GTG tRFLeu-CAG	tRF-3	associate with Ago2 and downregulate target genes by transcript cleavage	[38]
ovarian cancer	tRF5-Glu	tRF-5	Increased tRF5-Glu inhibits the proliferation of ovarian cancer cells.	[39]
	tRF-03357	tRF-5	tRF-03357 promotes cell proliferation, migration, and invasion.	[40]
osteosarcoma	tiRNA^Ala^ tiRNA^Cys^	tiRNA-5	Inhibit protein synthesis and trigger the phospho-eIF2α-independent assembly of stress granules	[41]
	tRF^Val^	tRF-5	tRF^Val^ induces the assembly of cytoprotective stress granules.	[41]
chronic lymphocytic leukemia	ts-101, ts-53	tRF-1	ts-53 targets the 3′-UTR of TCL1, a key oncogene in the development of aggressive CLL. ts-101 and ts-53 associate with PiwiL2, an essential protein involved in silencing of transposons.	[42]
	ts-46, ts-47	tRF-1	potential tumor suppressors	[43]
	i-tRF-GlyCCC	i-tRF	predict poor overall survival	[44]
	i-tRF-GlyGCC	i-tRF	prognostic biomarker	[45]
	ts-43, ts-44	tRF-5	tumor suppressors	[46]
head and neck squamous cell carcinoma	5′-tiRNA^Ala^ 5′-tiRNA^Cys^ 5′-tiRNA^Tyr^	tiRNA-5	significantly increase	[47]
uveal melanoma	tRF-22-BP4MJYSZH tRF-21-45DBNIB9B	i-tRF	associated with metastasis and patient survival	[48]
gastric cancer	tRF-3019a	tRF	tRF-3019a overexpression enhances gastric cancer cell proliferation, migration, and invasion	[49]
testicular germ cell tumor	tRF (20 nt)	tRF	Associated with cancerdevelopment and progression	[26]

**Table 2:** Roles of tRNA-derived fragments and tRNA halves in cancer drug resistance

Cancer	tRFs and tiRNAs	Findings	References
breast cancer	tDR-0009 tDR-7336	tDR-0009 and tDR-7336 can maintain cell response to IL-6, which participates in multidrug resistance by activating the JAK/STAT3, PI3K/Akt, and Ras-MAPK pathways.	[13, 50]
	tDR-0124 tDR-11,898	tDR-0124 and tDR-11,898 are involved in chemoresistance	[13]
	tRF-30-JZOYJE22RR33 tRF-27-ZDXPHO53KSN	trastuzumab resistance	[12]
	tDR-5334	tamoxifen resistance	[16, 51]
	tDR-4733	tDR-4733 mediates acquired resistance to HER2 inhibitors through the PI3K/AKT/mTOR signaling pathway.	[52]
	5′-tiRNA^Val^	5′-tiRNA^Val^ can directly bind to FZD3 and inhibit the FZD3-mediated Wnt/β-catenin pathway, which is related to tamoxifen and doxorubicin resistance.	[18]
	tRF-2 derived from tRNA^Glu^, tRNA^Asp^, tRNA^Gly^ and tRNA^Tyr^	tRFs increase chemosensitivity of various tumors by replacing 3′-UTR from YBX1.	[23, 53]
	ts-46, ts-47	ts-46 and ts-47 are upregulated by PIK3CA and KRAS mutations, respectively. These two mutations are involved in the resistance of breast cancer cells to lapatinib.	[26]
lung cancer	tRF-Leu-CAG	tRF-Leu-CAG may be related to AURKA. AURKA overexpression induces gefitinib and cisplatin resistance.	[27, 54, 55]
	ts-101, ts-46, ts-47	Mediate multidrug resistance	[25]
pancreatic cancer	tRF-1391	Target genes of tRF-1391 are mainly concentrated in the PI3K/Akt pathway, which is related to doxorubicin resistance.	[28, 56]
colorectal cancer	tRF/miR-1280	tRF/miR-1280 binds to 3′-UTR of JAG2, thus inhibiting Notch/Gata and miR-200b signaling. JAG2, Notch, Gata, and miR-200b are related to drug resistance.	[29]
	tiRNA-Tyr-GTA tRF-Gln-CTG tRF-Leu-TAG	chemoresistance	[30]
ovarian cancer	tRF-03357 tRF-03358	tRFs are involved in the MAPK, FoxO, and Wnt pathways and affect sensitivity to cisplatin.	[40]
chronic lymphocytic leukemia	ts-101, ts-53, ts-46, ts-47	unclear	[25]

### Mechanisms of tRFs and tiRNAs in cancer drug resistance

Drug resistance occurs when the therapeutic efficacy of a drug decreases. Drug resistance is an obstacle to cancer treatment and patient survival. The tRFs and tiRNAs can replace eukaryotic translation initiation factor 4G (eIF4G) that binds to mRNA, thus inhibiting protein translation [41]. The downregulation of eIF4G increases doxorubicin sensitivity by inhibiting the expression of adenosine triphosphate-binding cassette (ABC) transporter in breast cancer cells [60]. ABC transporter can efflux anticancer drugs across cell membranes, which is associated with drug resistance of many solid tumors [13, 61]. The inhibition of eIF4F complex, including eIF4E, eIF4G, and eIF4A, enhances the sensitivity of various anticancer drugs, such as cisplatin sensitivity in non-small cell lung cancer (NSCLC) [62], trastuzumab and tamoxifen sensitivity in breast cancer [63, 64], and enzalutamide and bicalutamide sensitivity in castration-resistant prostate cancer (CRPA) [65]. In addition, tRFs and tiRNAs can promote the assembly of stress granules (SGs) under stress conditions [40]. SGs allow cells to recruit and protect mRNAs during stress [66]. SGs are related to drug resistance [67, 68]. The elimination of SG sequestration is associated with acquired drug resistance [69]. The assembly of SGs makes glioblastoma resistant to bortezomib, and thus, the drug is unable to inhibit angiogenesis [66].

### Mechanisms of breast cancer resistance related to tRFs and tiRNAs

Breast cancer ranks second among the causes of female cancer-related deaths [70]. Chemotherapy resistance causes high mortality of patients with breast cancer [71]. The exact mechanisms of chemoresistance have not yet been fully elucidated. Herein, we summarize the mechanisms by which tRFs play a role in resistance to breast cancer therapy.

**Fig. 2:**
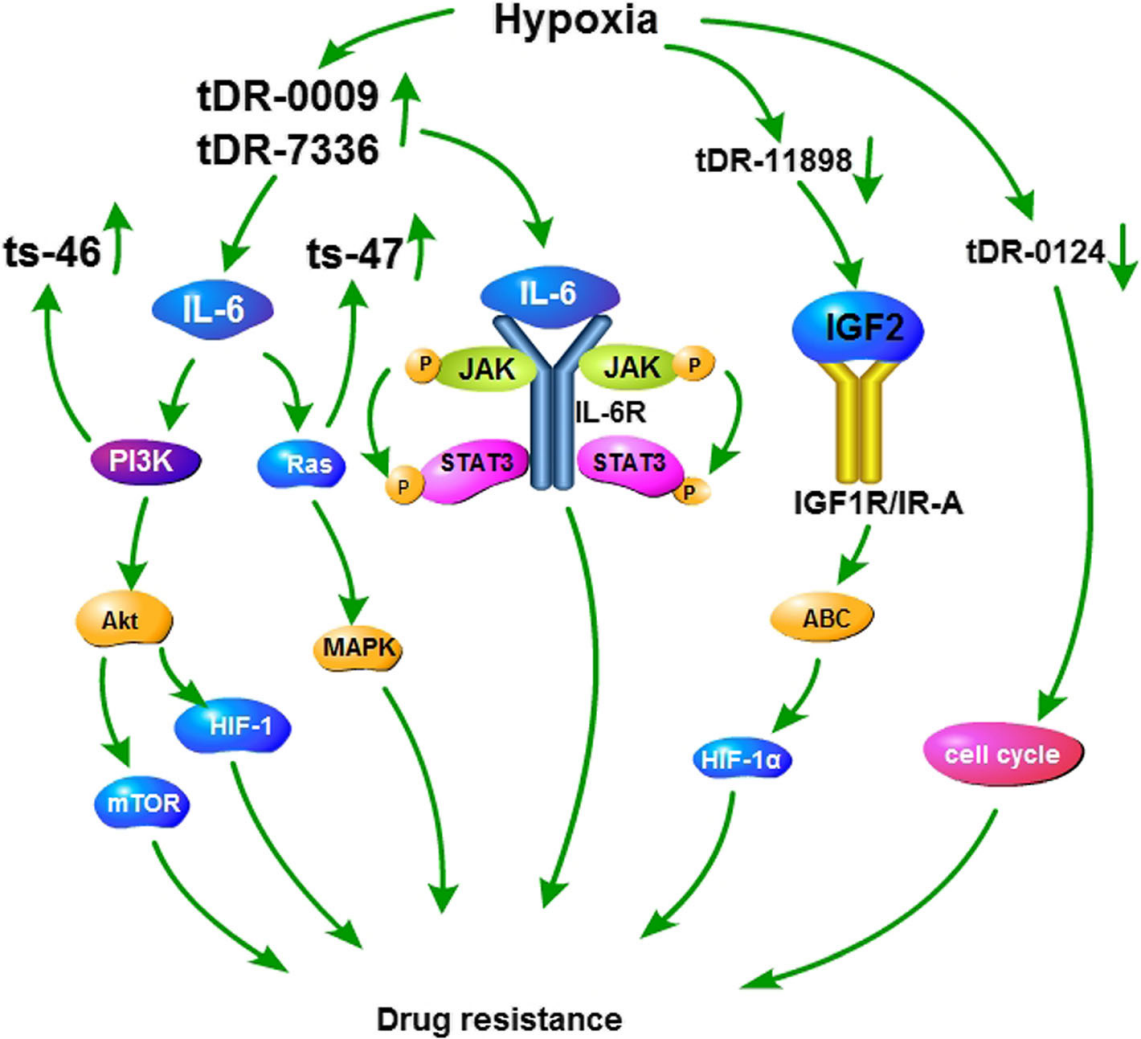
Mechanisms of tDR-0009, tDR-7336, tDR-0124, tDR-11,898, ts46, and ts47 in breast cancer resistance. tDR-0009 and tDR-7336 are upregulated, while tDR-0124 and tDR-11,898 are downregulated in resistance of triple-negative breast cancer to doxorubicin. tDR-0009 and tDR-7336 can maintain cell response to IL-6. IL-6 participates in multidrug resistance by activating the JAK/STAT3, PI3K/Akt/mTOR, and Ras-MAPK pathways. AKT can induce HIF-1 and mediate drug resistance. ABC transporter plays an important role in breast cancer resistance. Target genes of tDR-0124 are related to cell cycle regulation, which are involved in chemoresistance. IGF2 is one of the proteins that interact most frequently with the target genes of tDR-11,898. The binding of IGF2 to IGF1R/IR-A activates the downstream signaling cascade and eventually stimulates the expression of ABC transporters. ts-46 and ts-47 are upregulated by PIK3CA and KRAS mutations, respectively. Both mutations are related to the development of breast cancer chemoresistance

Resistance is common in triple-negative breast cancer (TNBC) [13]. Hypoxia is a characteristic of the tumor microenvironment and is related to tumor aggressiveness, metastatic potential, and chemoresistance [72]. Hypoxia can also induce tRFs. Hypoxia promotes chemoresistance to TNBC treatment in various ways. It can hinder drug penetration, affect cytotoxicity of drugs, induce breast cancer stem cell (CSC) phenotype, and regulate tumor immunity [72]. However, few studies have linked hypoxia-induced tRFs to drug resistance. Cui et al. studied the role of hypoxia-induced tRFs in doxorubicin resistance during TNBC treatment and found that tDR-0009 and tDR-7336 were significantly upregulated, while tDR-0124 and tDR-11,898 were downregulated. Gene ontology (GO) analysis indicates that tDR-0009 and tDR-7336 can maintain cell response to interleukin (IL)-6 [13]. IL-6 participates in multidrug resistance by activating the Janus kinase (JAK)/Signal transducer and activator of transcription 3 (STAT3), phosphoinositide 3-kinase (PI3K)/Protein Kinase B (Akt), and Ras-Mitogen-activated protein kinase (MAPK) pathways [50]. STAT3 mediates TNBC resistance through the NF-κB pathway, Bcl-2-associated x protein (Bax), and the TNFRSF1A gene [59, 73, 74]. In TNBC immunotherapy, STAT3 enhances PD-L1 expression, thus weakening the response to anti-PD-L1 therapy [75]. Furthermore, IL-6 can increase HIF-1α expression by activating STAT3. HIF-1α mediates the expression of P-glycoprotein (P-gp) and Multidrug Resistance Protein 1 (MRP1) [50]. In conclusion, tDR-0009 and tDR-7336 may participate in the drug resistance of TNBC by regulating the activation of STAT3 phosphorylation [13]. Target genes of tDR-0124 are related to cell cycle regulation, while target genes of tDR-11,898 participate in the regulation of RNA polymerase II promoter transcription and RNA biosynthesis, which are involved in chemoresistance [13]. Insulin-like growth factor 2 (IGF2) is one of the proteins that interact most frequently with the target genes of tDR-11,898 [13]. The binding of IGF2 to insulin-like growth factor receptor 1 (IGF1R)/insulin receptor A (IR-A) activates the downstream signaling cascade and eventually stimulates cell growth and expression of ABC transporters [76, 77]. These data illustrate that tRF-0009, tDR-7336, tDR-0124 and tDR-11,898 may play a role in hypoxia-induced chemoresistance through multiple mechanisms (Fig. 2).

Sun et al. investigated tRFs related to trastuzumab resistance in human epidermal growth factor receptor-2 (HER2)-positive breast cancer [12]. tRF-30-JZOYJE22RR33 and tRF-27-ZDXPHO53KSN were overexpressed in trastuzumab-resistant patients. These two tRFs may lead to trastuzumab resistance by regulating the expression product of target genes or competing with mRNAs for binding to RNA-binding proteins [12]. They are intervention targets for predicting trastuzumab resistance in the treatment of breast cancer.

Huang et al. reported that tDR-7816, tDR-5334, and tDR-4733 were downregulated in nontriple-negative breast cancer patients compared to healthy people [16]. GO and KEGG pathway analyses indicate that the target genes of tDR-5334 are involved in the glycosylation process. Abnormal glycosylation is involved in the activation of oncogenic signaling pathways and induces cancer metastasis [16]. N-glycosylation of β1-integrin in epithelial-to-mesenchymal transition (EMT) partly explained the reason for trastuzumab resistance in HER2-positive breast cancer [78]. Glycosylation is a non-negligible multidrug resistance mechanism. The analysis of target genes of tDR-5334 revealed that STAT1 is the central protein in the protein-protein interaction (PPI) network [16]. STAT1 regulates ERα transcription and ERα signaling and is associated with tamoxifen resistance [51]. The analysis of target genes of tDR-4733 showed that tDR-4733 is involved in lipid metabolism and cell cycle regulation [16]. Changes in lipid metabolism mediate the development of acquired resistance to HER2 inhibitors through the PI3K/AKT/mTOR signaling [52]. The central protein cyclin B1 is a key part of cell cycle that causes cells to undergo mitosis [16]. Sabbaghi et al. reported that trastuzumab-emtansine-induced cyclin B1 deficiency mediates drug resistance in HER2-positive breast cancer [79]. In addition, hypoxia can inhibit the expression of cyclin B1 in MCF-7 cells. Overexpression of cyclin B1 enhances paclitaxel sensitivity. Therefore, under hypoxic conditions, downregulated cyclin B1 can further promote paclitaxel resistance [80].

Mo et al. reported that the expression of 5′ fragment of tRNA-Val-CAC (5′-tiRNA^Val^) was reduced in breast cancer [18]. The 5′-tiRNA^Val^ regulated cell proliferation, migration, and invasion in breast cancer. Frizzled homolog 3 (FZD3), β-catenin, c-myc, and cyclin D1 were downregulated, while *Adenomatous Polyposis Coli* (APC) was upregulated in cells overexpressing 5′-tiRNA^Val^. The FZD3 protein is mapped to chromosome 8p21, a major component of the Wnt signaling pathway, which is involved in regulating early neural development [81]. The 5′-tiRNA^Val^ can directly bind to FZD3 and inhibit FZD3-mediated Wnt/β-catenin pathway [18], which is related to tamoxifen and doxorubicin resistance [82, 83]. In addition, the downregulation of FZD3 significantly reduces the expression of cyclin D1 and c-myc [18]. C-myc can confer chemoresistance in breast cancer cells [84, 85]. Seventy percent of patients with sporadic breast cancer lose APC because of mutation or hypermethylation [86, 87]. The loss of APC results in resistance to doxorubicin and cisplatin by STAT3, EGFR, NOTCH, and Hedgehog signaling [88–90]. The STAT3 signaling pathway mainly plays a role in APC-mediated resistance to doxorubicin, but not to cisplatin [71]. To summarize, we speculate that 5′-tiRNA^Val^ plays a role in breast cancer chemoresistance through FZD3, β-catenin, c-myc, and APC.

In breast cancer, tRNAGlu, tRNAAsp, tRNAGly, and tRNATyr are exposed to stress and are enzymatically cleaved to produce tRFs [23]. The induction of these tRFs inhibits the stability of oncogenic transcripts by replacing 3′-UTR from the RNA-binding protein Y Box-Binding Protein 1 (YBX1), thereby inhibiting metastasis [23]. YBX1 is a versatile RNA-binding protein with a variety of interacting partners. Genetic inactivation of YBX1 leads to embryonic lethality [91]. YBX1 is overexpressed in multiple cancers [53] and enhances chemoresistance in melanoma [92], embryonal rhabdomyosarcoma [93], NSCLC [94], prostate cancer [95], gastric cancer [96], breast cancer [97], neuroblastoma [53], hepatocellular carcinoma [98], ovarian carcinoma [99], bladder cancer [100], diffuse large B-cell lymphoma [101], prostate cancer [102], chordomas [103], and esophageal squamous cell carcinoma (ESCC) [104]. YBX1 promotes cancer chemoresistance by upregulating ABC transporters related to multidrug resistance [53, 94, 97, 104, 105]. Overexpression of YBX1 confers resistance to mitoxantrone by cell adhesion mechanisms in diffuse large B-cell lymphoma [101]. In addition, YBX1 also transcriptionally activates the expression of drug resistance-related genes MVP/LRP, TOP2A, CD44, CD49f, BCL2, and MYC [106]. In summary, tRFs may increase chemosensitivity of various tumors by replacing 3′-UTR from YBX1.

In addition, Corce M et al. reported that ts-46 and ts-47 were upregulated by PIK3CA and KRAS mutations, respectively. These two mutations were involved in the resistance of breast cancer cells to lapatinib [26].

Chemoresistance causes high mortality of patients with breast cancer. The mechanisms by which tRFs induce chemoresistance in breast cancer can be applied to develop therapies to overcome drug resistance and prolong survival.

### Mechanisms of lung cancer resistance related to tRFs and tiRNAs

**Fig. 3:**
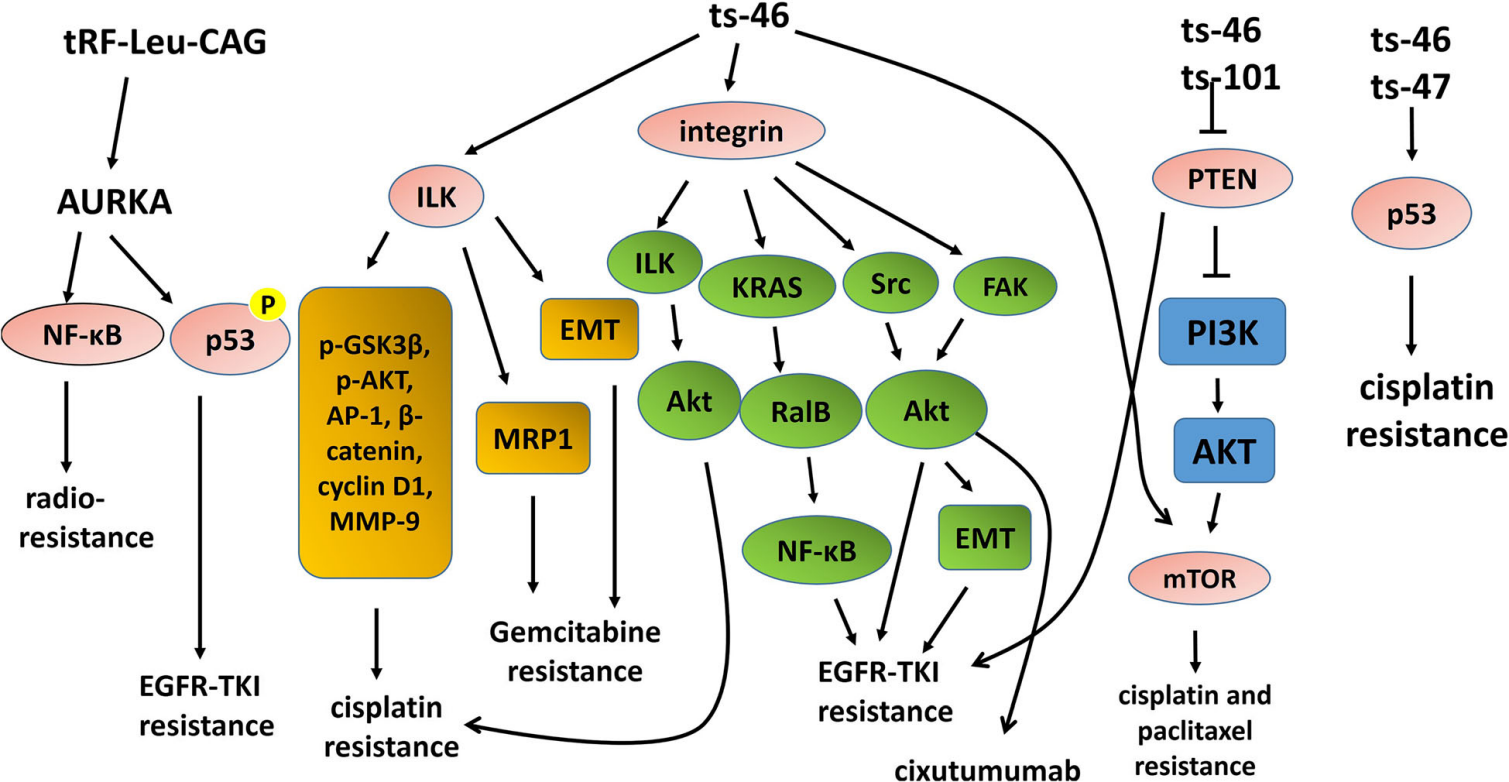
tRF-Leu-CAG, ts-101, ts-46, and ts-47 are involved in drug resistance of lung cancer cells. Knockdown of tRF-Leu-CAG downregulates AURKA. AURKA overexpression induces gefitinib resistance by downregulating p53. The AURKA/NF-ĸB pathway is related to radio-resistance. ts-46 is associated with excessive activation of ILK, integrin, and mTOR signaling. Both ts-46 and ts-101 are correlated with the inhibition of PTEN signaling. RNA silencing of ILK enhances cisplatin sensitivity by regulating p-GSK3β, p-AKT, AP-1, β-catenin, cyclin D1, and MMP-9. Integrin activates Akt, which determines resistance to cisplatin. Integrin mediates EGFR-TKIs resistance through EMT, the KRAS-RalB-NF-κB pathway, and the Src/Akt pathway. Integrin/Src/Akt in NSCLC cells confers resistance to cixutumumab. PI3K/AKT/mTOR signaling promotes chemoresistance. ts-46 and ts-47 can affect the p53 pathway, which mediates cisplatin resistance in NSCLC

Lung cancer has the highest mortality rate among all tumors. The 5-year survival rate is 19% [70]. Among all lung cancers, NSCLC accounts for more than 80% [107]. Epidermal growth factor receptor tyrosine kinase inhibitors (EGFR-TKIs) usually produce incomplete response within 9–12 months of treatment and then develop resistance. Shao et al. found that tRF-Leu-CAG was significantly upregulated in advanced NSCLC and promoted cell proliferation. Knockdown of tRF-Leu-CAG downregulated Aurora-A kinase (AURKA) in H1299 cells, indicating that tRF-Leu-CAG may be related to AURKA [27]. AURKA is a member of the Aurora kinase family of serine/threonine kinases and is involved in cell mitosis [108]. AURKA phosphorylates p53 at Ser315, resulting in its ubiquitination by Mdm2 and proteolysis [109]. In HCC827 cells, AURKA overexpression induced gefitinib resistance by downregulating the p53 signaling pathway [54]. The combination of EGFR-TKIs and AURKA inhibitors can suppress resistance [110]. Furthermore, in vitro data showed that AURKA overexpression was related to cisplatin resistance [55]. In addition to targeted therapy, the AURKA/NF-ĸB pathway is related to radio-resistance in docetaxel-resistant lung adenocarcinoma [111]. These data indicate that tRF-Leu-CAG may become a potential therapeutic target to reverse drug resistance in NSCLC. (Fig. 3).

Balatti et al. found that ts-101, ts-53, ts-46, and ts-47 were downregulated in lung cancer [25]. A functional enrichment study showed that ts-46 was associated with excessive activation of integrin-linked kinase (ILK) signaling, integrin signaling, platelet-derived growth factor (PDGF) signaling, sphingosine-1-phosphate (S1P) signaling, and mTOR signaling, while ts-101 was related to the overactivation of S1P signaling and glutamate receptor signaling. Both ts-46 and ts-101 were correlated with the inhibition of phosphatase and tensin homolog (PTEN) and ceramide signaling [25]. ILK is a serine/threonine protein phosphatase and related to tumor growth and metastasis [112]. RNA silencing of ILK enhances cisplatin sensitivity in lung adenocarcinoma by regulating the downstream genes p-GSK3β, p-AKT, AP-1, β-catenin, cyclin D1, and MMP-9 [113]. ILK participates in the resistance of lung cancer to gemcitabine through EMT and MRP1 [112]. Integrins are transmembrane proteins that can control aggressive behaviors of tumors [114]. Integrin stimulates ILK by binding to extracellular matrix components and further activates the protein kinase B/Akt. Akt activity determines resistance to cisplatin [113]. Integrin mediates EGFR-TKIs resistance through EMT, the KRAS-RalB-NF-κB pathway, and the Src/Akt pathway [115–118]. The integrin α(v)β_3_/Src/Akt pathway confers NSCLC cells resistance to anti-IGF1R monoclonal antibody cixutumumab [119]. PDGF/PDGFR may be one of the mechanisms of excision repair cross-complementing 1 (ERCC1)-mediated cisplatin resistance [120]. PI3K/AKT/mTOR signaling promotes chemoresistance in lung cancer [121–123]. PTEN is a major negative regulator of the PI3K pathway [25]. PTEN inactivation is related to EGFR-TKIs resistance [124, 125]. Silencing of PTEN induces resistance to cisplatin and paclitaxel through the PI3K/Akt pathway in NSCLC [126–128]. ts-46 and ts-47 can counteract carcinogenic effects of KRAS mutations and simultaneously positively affect the p53 pathway [25]. The P53 pathway mediates cisplatin resistance in NSCLC [129, 130]. These data indicate that ts-101, ts-46, and ts-47 may be involved in drug resistance of lung cancer cells. (Fig. 3).

### Mechanisms of pancreatic cancer resistance related to tRFs and tiRNAs

Pancreatic cancer (PC) has a high mortality rate, and the 5-year survival rate of patients with PC is only 8% [131]. Jin et al. reported that tRF-1391 and AS-tDR-000064 were associated with PC [28]. The KEGG pathway analysis showed that target genes of tRF-1391 were mainly concentrated in the PI3K/Akt pathway and 18 target genes of AS-tDR-000064 were related to resistance to EGFR-TKIs [28]. Metformin mediates PI3K/Akt/mTOR signaling, thereby enhancing the sensitivity of gemcitabine treatment in PC [132]. Furthermore, the PI3K/AKT signaling pathway is related to doxorubicin resistance in PC [56]. The roles of tRFs in drug resistance of PC need further research.

### Mechanisms of colorectal cancer resistance related to tRFs and tiRNAs

Colorectal cancer (CRC) ranks third in morbidity and mortality among all tumors [70]. Chemoresistance leads to a low response rate to chemotherapy in advanced CRC [133]. Huang et al. reported that tRF/miR-1280 expression was reduced in CRC. tRF/miR-1280 was derived from tRNALeu and pre-miRNA. In addition, the miR-200b level decreased and Jagged-2 (JAG2), Gata1, Gata3, and zinc finger E-box binding homeobox 1 (Zeb1) levels increased in CRC [29]. JAG2 is a Notch ligand and is involved in tumor initiation and maintenance [134]. Gata factors are zinc finger DNA-binding proteins that control tissue development through the activation or repression of transcription [135]. tRF/miR-1280 binds to 3′-UTR of JAG2, thus inhibiting Notch/Gata and miR-200b signaling. The inactivation of Notch signal mediated by tRF/miR-1280 inhibits CSC phenotype through transcriptional repressing of Gata1/3 and miR-200b and further inhibits cell proliferation and metastasis in CRC [29]. Ectopic expression of JAG2 mediates drug resistance in CRC. JAG2 regulates the sensitivity of CRC cells to chemotherapeutic drugs through p21 [136]. Notch signaling regulates cell proliferation, differentiation, and apoptosis [137] and is related to chemoresistance of colorectal CSCs [138]. Notch-1 mediates regorafenib resistance [137]. Reversing Notch signaling sensitizes CRC cells to 5-fluorouracil (5-FU) and irinotecan [139]. In addition, tRF/miR-1280 downregulated the expression of Gata1 and Gata3 through the Notch pathway. Gata binds to miR-200b to inhibit transcription initiation. Inhibition of Gata1 increases miR-200b expression and reduces EMT [29]. EMT and miR-200b are involved in chemoresistance of CRC [140, 141]. Furthermore, Zeb1 and miR-200b regulate each other negatively. The genes ubiquitin-specific peptidase 17, chromodomain helicase DNA-binding protein 1-like, and double homeobox 4 are associated with DNA damage response. The inhibitory effect of Zeb1 on these three genes induces drug resistance [142]. In summary, tRF/miR-1280 may be involved in chemotherapy resistance of CRC. Drugs that targeting tRF/miR-1280 may reverse drug resistance in future treatment and prolong survival.

Wang et al. reported that tiRNA-Tyr-GTA and tRF-Gln-CTG were upregulated, while tRF-Leu-TAG was downregulated in CRC [30]. The tiRNA-Tyr-GTA, tRF-Gln-CTG, and tRF-Leu-TAG are tiRNA-5, tRF-5c, and tRF-5a with lengths of 30, 29, and 16 nt, respectively. The GO and KEGG pathway analysis indicated that the functions of tiRNA-Tyr-GTA targets are mainly negative regulation of epithelial cell apoptotic and peroxisome proliferator activated-receptor (PPAR) pathway [30]. PPAR is a ligand-activated transcription factor and is involved in regulating cancer progression [143]. The PPAR pathway is related to radiation resistance in CRC [144]. Inhibition of PPARα expression confers resistance to hydroxycamptothecin [145]. PPARδ confers resistance to PPARγ-induced apoptosis in CRC through mediation of survivin and caspase-3 [146]. In addition, tRF-Gln-CTG enhances the negative regulation of c-Jun N-terminal kinase (JNK) cascade and choline metabolism, while tRF-Leu-TAG enhances MET function [30]. Silencing of the JNK1 gene can dephosphorylate c-Jun and reduce transport of the G2 subfamily of ABC transporters (ABCG2). The JNK1/c-jun pathway is related to ABCG2-mediated multidrug resistance in CRC [147]. JNK activation can confer resistance to 5-FU in CRC patients with p53 mutation by inducing Bcl-2 phosphorylation [148]. Taken together, tiRNA-Tyr-GTA, tRF-Gln-CTG, and tRF-Leu-TAG may be involved in chemoresistance of CRC.

### Mechanisms of ovarian cancer resistance related to tRFs and tiRNAs

Ovarian cancer (OC) is the fourth most common gynecological malignant tumor with high drug resistance [149]. High grade serous ovarian cancer (HGSOC) accounts for 75% of OC cases. Zhang et al. identified 27 differentially expressed tRFs, such as tRF-03357 and tRF-03358, from serum samples of patients with HGSOC and healthy controls. These 27 tRFs are involved in the MAPK, FoxO, and Wnt pathways [40]. Most patients with advanced OC develop treatment resistance [150]. MAPK is associated with acquired chemoresistance of OC [151]. The C-KIT/MAPK/MEK pathway establishes a link between platinum resistance and CSC phenotype in OC [152]. The Ras-MAPK/Erk-ETS1-ELK1/CFTR1 axis confers resistance to cisplatin [153]. The ERK/MAPK pathway promotes EMT phenotype, which is accompanied by increased cisplatin resistance [154]. The p38 MAPK activation upregulates p-glycoprotein expression, which enhances drug efflux, thereby inducing OC cell resistance to paclitaxel [155]. In addition, the activation of Wnt and its downstream molecules Wnt5a, β-catenin, c-Myc, and cyclin D1 upregulates the glycolysis level, thereby inducing cisplatin resistance in OC [156]. Fukumoto et al. showed that N6-methyladenosine of Frizzled family receptor 10 mRNA promoted resistance to poly (ADP)-ribose polymerase inhibitors (PARPi) by upregulating the Wnt/β-catenin pathway in BRCA-mutated epithelial ovarian cancer (EOC) [157]. FoxO proteins have been reported to affect the effectiveness of anticancer drug treatment. Reduced FoxO1 expression is related to resistance to cisplatin [158]. To summarize, these differentially expressed tRFs affect the sensitivity of OC to chemotherapy, especially to cisplatin.

In addition, Zhou et al. reported that tRF5-Glu was expressed in OC. The tRF5-Glu binds to breast cancer antiestrogen resistance 3 (BCAR3) and downregulates its expression. BCAR3 and tRF5-Glu contribute to heterogeneity of OC [39]. Overexpression of BCAR induces antiestrogen resistance in breast cancer cells; however, it remains unclear whether BCAR has a similar effect in OC resistance [159, 160].

### Mechanisms of chronic lymphocytic leukemia resistance related to tRFs and tiRNAs

Balatti et al. found that ts-101, ts-53, ts-46, and ts-47 were downregulated in chronic lymphocytic leukemia (CLL) [25]. As mentioned before, ts-46 is associated with integrin and ceramide signaling. ts-101 is correlated with ceramide signaling inhibition [25]. The combination of fibronectin and α4β1 integrin induces fludarabine resistance in B-cell CLL by upregulating Bcl-xL [161]. Ceramide metabolism is related to fludarabine resistance in CLL [162]. In addition, the downregulation of ts-53 increases the TCL1 expression level, leading to the progression of CLL [42]. However, the role of TCL1 in chemoresistance of CLL remains unclear.

### Techniques for studying tRFs and tiRNAs

In the process of studying tRF and tiRNA, it is quite important to differentiate between tRFs and randomly degraded fragments. By using specific amplification primers, tRFs and tiRNAs can be specifically detected by quantitative reverse transcription-polymerase chain reaction (qRT-PCR). The expression of tRFs and tiRNAs can also be detected by northern blot [1]. Splinted ligation assay can measure the levels of tRFs and tiRNAs. The assay depends on ligation between the 3′-end of tRF and the 5′-end of a ligation oligo, mediated through accurate annealing to a bridge oligo [31]. Microarray chips have been designed to study the expression of tRFs (shorter than 16 bp) in normal and tumor tissues [43]. Researchers have also established some databases to manage tRFs, such as trfdb (http://genome.bioch.virginia.edu/trfdb) [5], tRF2Cancer (http://rna.sysu.edu.cn/tRFfinder/) [58], YM500v3 database (http://ngs.ym.edu.tw/ym500/) [163], tRFexplorer (https://trfexplorer.cloud/) [164], and MINTbase v2.0 (http://cm.jefferson.edu/MINTbase/) [165]. Mitochondrial and Nuclear TRF mapping (MINTmap; https://github.com/TJU-CMC-Org/MINTmap/) is a software package for rapid and detailed identification of tRFs in short RNA-seq datasets. MINTmap can explicitly calculate and report raw and standardized abundances of discovered tRFs [1]. New technologies have led to breakthroughs in the exploration of tRFs and tiRNAs.

## Conclusion

It is very common for tumors to acquire resistance to anticancer drugs. There is an urgent need to elucidate molecular mechanisms of cancer chemoresistance. This review outlines the classification of tRFs and tiRNAs, their roles in cancers, the mechanisms by which they play a role in cancer drug resistance, and the techniques for studying them. Their high equivalent and stability in body fluids, and differential expression between cancer patients and healthy controls open up broad prospects to develop screening, diagnostic, and prognostic biomarkers and targeted anticancer drugs that enhance the sensitivity of chemotherapeutic drugs. As the field is still in its infancy, it is a major challenge to fully understand the mechanism network of tRFs and tiRNAs in drug resistance. Overall, tRFs and tiRNAs give hope for cancer treatment, but further studies are required before clinical application. More work needs to be done to study broader and deeper mechanisms of tRFs and tiRNAs in drug resistance and the crosstalk between various signaling pathways.

## Data Availability

Not applicable.

## Abbreviations

tRFs: tRNA-derived Fragments
tiRNAs: tRNA halves
tRNA: transfer RNA
nt: Nucleotide
tiRNA-5: 5′-tRNA half
tiRNA-3: 3′-tRNA half
eIF4G: Eukaryotic translation initiation factor 4G
ABC: Adenosine triphosphate-binding cassette
NSCLC: Non-small cell lung cancer
CRPA: Castration-resistant prostate cancer
SGs: Stress granules
TNBC: Triple-negative breast cancer
CSCs: Cancer stem cells
GO: Gene Ontology
KEGG: Kyoto Encyclopedia of Genes and Genome
IL: Interleukin
JAK: Janus kinase
STAT3: Signal transducer and activator of transcription 3
PI3K: Phosphoinositide-3-kinase
Akt: Protein Kinase B
MAPK: Mitogen-activated protein kinase
Bax: Bcl-2-associated x protein
mTOR: Mammalian target of rapamycin
P-gp: P-glycoprotein
MRP1: Multidrug Resistance Protein 1
Hh: Hedgehog
ER: Estrogen receptor
IGF2: Insulin-like growth factor 2
IGF1R: Insulin-like growth factor receptor 1
IR-A: Insulin receptor A
HER2: Human epidermal growth factor receptor-2
EMT: Epithelial-to-mesenchymal transition
FZD3: Frizzled homolog 3
APC: Adenomatous polyposis coli
TIC: Tumor initiating cell
YBX1: Y Box-Binding Protein 1
ESCC: Esophageal squamous cell carcinoma
EGFR-TKIs: Epidermal growth factor receptor tyrosine kinase inhibitors
ILK: Integrin-linked kinase
PDGF: Platelet-derived growth factor
S1P: Sphingosine-1-phosphate
PTEN: Phosphatase and tensin homolo
PC: Pancreatic cancer
CRC: Colorectal cancer
Zeb1: Zinc finger E-box binding homeobox 1
EMT: Epithelial-mesenchymal transition
PPAR: Peroxisome proliferator activated-receptors
JNK: c-Jun N-terminal kinase
ABCG2: G2 subfamily of ABC transporters
OC: Ovarian cancer
HGSOC: High-grade serous ovarian cancer
BCAR3: Breast Cancer Anti-Estrogen Resistance 3
CLL: Chronic lymphocytic leukemia
